# Community-level determinants of RTS,S/AS01 vaccine acceptance in a hyperendemic Ghanaian Region: A Bayesian multilevel analysis from Kpando Municipality, Ghana

**DOI:** 10.1371/journal.pgph.0005436

**Published:** 2025-11-21

**Authors:** Precious Kwablah Kwadzokpui, Kenneth Ablordey

**Affiliations:** 1 Medical Laboratory Department, Ho Teaching Hospital, Ho, Ghana; 2 Department of Epidemiology and Disease Control, School of Public Health, University of Ghana, Accra, Ghana; 3 Department of Medical Laboratory Science, School of Allied Health Sciences, University of Health and Allied Sciences, Ho, Ghana; University of Alabama at Birmingham, UNITED STATES OF AMERICA

## Abstract

The successful scale-up of Ghana’s RTS,S/AS01 malaria vaccine depends on understanding community-level variation in caregiver acceptance. This study investigates vaccine acceptance and its predictors in a hyperendemic municipality bordering Volta Lake, where geographic and contextual disparities may hinder equitable implementation. A weighted, community-based cross-sectional survey was conducted from November 2023 to January 2024 among 452 caregivers of children aged 6–59 months in Kpando Municipality. Data were collected via structured digital interviews and analyzed using Bayesian multilevel logistic regression to identify individual and contextual determinants, with spatial mapping to visualize geographic disparities. Overall vaccine acceptance was 89.9% (95% CI: 89.2–90.7), with sub-district variation from 41.1% in Agbenorhoe to 100% in several island and rural communities. Among children initiating vaccination, 6.0% received one dose, 17.6% two doses, 10.4% three doses, and 66.0% completed four doses. Booster completion (66.0%) significantly exceeded primary series completion (34.0%, p < 2 × 10 ⁻ ¹⁶). For initial acceptance, bush-surrounded households had higher odds (aOR = 2.69) while vaccine risk concern reduced acceptance (aOR = 0.32). For booster completion, higher household income (1500–1900 GHC: aOR = 3.19; 2000–2400 GHC: aOR = 2.75), older child age (1 year: aOR = 2.27; 2 years: aOR = 2.14), family/peer influence (aOR = 2.09), and perceived convenience (aOR = 1.58) were positive predictors, while bush-surrounded residence reduced odds (aOR = 0.26). Sub-district-level factors explained 85% of variance (ICC = 0.85). Despite high acceptance, sharp spatial disparities and uneven dose completion persist. To ensure equitable coverage as Ghana scales up RTS,S/AS01, interventions must both address initial hesitancy in low-acceptance areas and improve primary series retention by reducing economic barriers and enhancing service convenience in rural communities.

## Introduction

Malaria persists as a leading cause of global morbidity and mortality, with almost 263 million estimated cases and 597,000 deaths reported in 2023. The World Health Organization (WHO) African Region bears the overwhelming share of this burden, accounting for roughly 94% of cases (≈246 million) and 95% of deaths (≈569,000), where children under five contribute the vast majority of deaths. Since 2020, global malaria cases have risen again, and between 2019 and 2023 the WHO African Region experienced an increase of approximately 23 million estimated cases and 24,000 deaths [1]. In 2023, the global incidence rate was 60.4 cases per 1,000 population at risk, with countries such as Ethiopia, Madagascar, Pakistan, Nigeria, and the Democratic Republic of the Congo contributing most to the year-on-year increase [1]. These trends emphasize the fragility of recent gains and the urgent need for novel tools, such as vaccination, to restore momentum toward malaria elimination. Ghana ranks among the top 18 highest-burden countries, contributing approximately 2.5% of global cases (1.9% of deaths), and continues to record malaria as the primary cause of under-five mortality [1,2]. Despite a 95% reduction in national malaria deaths from 2012–2022 [3], transmission remains intense in regions like Kpando Municipality, where malaria caused 5,827 cases in 2018 alone [4].

Current interventions, such as insecticide-treated nets (ITNs), rapid diagnostics, and artemisinin-based therapies, have resulted in declining prevalence but continue to face biological and operational challenges [2,5]. The 2021 WHO endorsement of the RTS,S/AS01 vaccine, the first malaria vaccine showing partial efficacy in Phase III trials [6], provides a transformative addition to existing tools. Pilot implementation in Ghana, Kenya, and Malawi under the Malaria Vaccine Implementation Programme (MVIP) demonstrated about 30% fewer severe malaria cases and nearly 13% lower all-cause child mortality in vaccinated areas compared with controls, based on WHO’s independent evaluations [7]. However, sustaining uptake across the full schedule proved challenging: coverage of the first three doses reached around 70–75%, but fewer than half of children received the fourth dose [1]. Broader challenges, such as suboptimal routine immunization coverage in low- and middle-income countries and lingering COVID-19 vaccine hesitancy in Ghana (≈45.9%) [8], pose additional threats to national scale-up. In response to these delivery barriers, global partners have intensified support for implementation. Notably, Gavi, the Vaccine Alliance, has established a dedicated Malaria Vaccine Programme to provide financing and technical assistance. By 2023, 20 countries had either introduced malaria vaccination or been approved to receive Gavi support, and by late 2024 additional introductions were underway across sub-Saharan Africa. With two WHO-recommended vaccines, RTS,S and R21, now available, supply is projected to keep pace with demand, creating new opportunities to overcome earlier access constraints and expand equitable coverage [1]. Variability in caregiver acceptance across SSA [9,10] underscores the importance of generating context-specific evidence.

Vaccine hesitancy in Ghana arises from a complex mix of sociocultural, informational, and systemic factors. Historical controversies, such as dengue vaccine trial suspensions [11] and polio immunization boycotts [12], have diminished trust. Studies in Upper East Ghana showed RTS,S coverage below 50% due to insufficient community engagement [13], while pre-implementation acceptance studies in Nigerian communities showed varying levels (68% to 98%) influenced by maternal education and safety perceptions [14,15]. In Kpando Municipality, a hyperendemic area where children under five years old constitute 10.05% of the population [15], no data exist on RTS,S acceptance, despite its high malaria burden and close proximity to Volta Lake, an environmental risk hotspot [16].

Kpando Municipality was included in the national rollout of the RTS,S/AS01 vaccine under the MVIP starting in May 2019. The vaccine was delivered through the existing Expanded Programme on Immunization (EPI) infrastructure of public health facilities. The initial schedule consisted of four doses: three primary doses administered at 6, 7, and 9 months of age (concurrently with pentavalent and measles-rubella vaccines), and a fourth dose at approximately 24 months. In alignment with a national policy change, the recommended age for the fourth dose was shifted to 18 months in January 2023 [17,18]. This revised schedule was implemented uniformly across all health facilities in the municipality simultaneously. The promotion of the vaccine was conducted through standardized national and regional channels, including health worker talks and community engagement sessions.

This study addresses key knowledge gaps by investigating the acceptance of the RTS,S malaria vaccine among caregivers of children aged 6–59 months in the Kpando municipality. It quantifies geospatial disparities, examines sociodemographic and environmental determinants, and applies Bayesian hierarchical modeling to identify predictive factors both for vaccine acceptance and booster dose (dose 4) update. The findings provide the first empirical evidence to guide the optimization of RTS,S vaccine delivery in Ghana’s lake-bordering communities and inform national deployment strategies within the context of ongoing regional malaria elimination efforts.

## Methods

### Study design

This study employed a community-based, cross-sectional design to examine factors associated with RTS,S/AS01 malaria vaccine acceptance among caregivers of children aged six months to under five years between 12^th^ November 2023 and 28^th^ January 2024.

### Study area

The study was conducted in the Kpando Municipality, located between latitudes 6° 20’ and 7° 05’ N and longitude 0° 17’ E in the Volta Region of Ghana [19]. It is bordered to the north by Biakoye District, to the east by Afadzato South and Hohoe Municipalities, to the south by North Dayi District, and to the west by the Volta Lake. The municipality covers a land area of 314.07 square kilometers and, according to the 2021 Population and Housing Census, has an estimated of approximately 58,552 people. The municipality’s health infrastructure comprises 17 healthcare facilities, including 10 community-based health planning and services (CHPS) compounds, three health centers, two private hospitals, and two private clinics. Notably, healthcare resources and access are disproportionately concentrated in the municipal capital, Kpando [4], a contextual factor that may influence vaccine rollout and acceptance patterns across the municipality.

### Study population and eligibility criteria

The study population included caregivers who were de jure residents of the Kpando municipality and had children aged between six months and five years. Caregivers were defined as individuals, regardless of gender, aged 15 years or older, who had primary responsibility for the day-to-day care of eligible children. Eligible caregivers had to meet three criteria: (1) they must have provided informed consent, (2) they must have a child aged six months or older but younger than five years, and (3) they must be de jure residents of Kpando. For this study, de jure residency was defined as either being a native of Kpando or having lived continuously in the municipality since before the introduction of the RTS,S vaccine through the Malaria Vaccine Implementation Programme (MVIP) in 2019. This definition aimed to ensure that attitudes and acceptance patterns reflected genuine exposure to the ongoing vaccination program. Caregivers who were too ill to participate or who were only on short-term visits to the area were excluded from the study.

### Sample size determination

Sample size estimation was performed using the Yamane formula for finite population sampling [20]. Based on the Aggregated data on 2021 Ghana Population and Housing Census, the population of children under five in the Kpando municipality was estimated at 5882. With a margin of error of 5%, the minimum required sample size was 375 participants. To account for potential nonresponse and data quality issues, a 10% upward adjustment was applied, resulting in a final minimum sample size of 412 caregivers.

### Sampling procedure

A three-stage multistage cluster sampling approach was implemented to select participants. In the first stage, a comprehensive list of all sub-districts and communities was obtained from the Kpando Municipal Health Directorate. From the five sub-districts, Agbenorxoe, Gbefi-Gadza, Kpando, Sovie-Kudzra, and Torkor Island, a random selection of communities was performed using random number generator in Microsoft Excel 365. Within the Torkor Island sub-district however, 18 communities were excluded due to limited accessibility; the municipal health staff classified 10 as requiring river crossing without reliable ferry service, and 8 as hard-to-reach. Three communities from Torkor Island, totaling 3,339 people and 835 households, were retained in the sample frame. In the second stage, a probability proportion to size (PPS) method was applied using the population of each selected community to allocate the number of respondents needed. In the third stage, within each selected community, households were visited to identify eligible children for recruitment into the study by trained research assistants. If multiple caregivers with eligible children were present, all were included. However, if a single caregiver had multiple eligible children, only the youngest child (aged 6–59 months) was considered to avoid duplication.

### Data collection instrument and process

The semi-structured questionnaire was developed and validated through face validation by two independent experts in malaria research and public health. They reviewed the instrument for content clarity, logical flow, language, and appropriateness for the study objectives. Based on their feedback, minor modifications were made before field deployment. Several items were adapted from previous malaria vaccine acceptance studies to ensure consistency with validated constructs [9,10,21,22]. Additionally, contextually relevant variables specific to the Kpando Municipality were developed and assessed.

Data were collected digitally using KoboCollect installed on Android-enabled smartphones. Research assistants were trained in survey administration, ethics, informed consent, and digital data entry. Data were collected in five modules: sociodemographics, household environment and health insurance, malaria knowledge and preventive behavior, RTS,S/AS01 vaccine awareness and perceptions, and contextual and psychosocial factors influencing vaccine acceptance. Child vaccination status was confirmed through inspection of page 52 of the maternal and child health record book. To ensure respondents differentiated the RTS,S malaria vaccine from other childhood vaccines, all questions in the relevant modules were prefaced with phrases such as ‘Specifically for the malaria vaccine, which is sometimes called the malaria injection...’. Interviewers were trained to use the local term for malaria vaccine (‘emu bi’) to ensure clarity and specificity. Caregivers with unverified child vaccination status were offered brief educational sessions at the end of the interview.

### Variable construction and coding

The main outcome variable was explicitly defined as acceptance of the RTS,S/AS01 malaria vaccine to distinguish it from general attitudes towards childhood vaccination. It was coded as a binary variable where “Yes” (1) indicated that the caregiver had accepted vaccination for their eligible child, and “No” (0) indicated refusal to accept or lack of consent. Vaccination status was initially self-reported by the caregiver and subsequently verified using entries in the maternal and child health record book. Additional vaccine dose variables were constructed. A dose completion variable recorded the total number of doses received (1–4). A categorical version of this variable distinguished between one, two, three, or all four doses. From this, a dose group variable was derived, separating children who completed only the primary series (doses 1–3) from those who also received the booster dose (dose 4). This enabled direct assessment of dose coverage patterns and booster completion. Data cleaning and transformation were carried out using the *dplyr* and *readxl* packages in R version 4.3.2, within the RStudio 2025.05.0 + 496 (“Mariposa Orchid”) environment.

Character variables were converted to categorical (factor) variables, and the raw sampling weights provided in the dataset were rescaled to reflect the estimated target population of children aged 6–59 months in the study area. Specifically, weights were normalized such that their sum equalled the known population size of 5,882, as provided by the GSS [19]. This normalization was done to ensure that weighted estimates were interpretable in absolute terms and appropriately representative of the under-five population within the Kpando Municipality.

The normalization was implemented using the following post-stratification weight normalization formula:


$$\omega_{i}^{norm}=\frac{\omega_{i}}{\sum_{i=\text{1}}^{n}\omega_{i}}\text{x}N$$

where $\omega_{i}$ is the original sampling weight for observation i,nis the total number of sampled observations, *N* = 5,882 is the estimated total population of under-five children and $\omega_{i}^{norm}$ is the normalized weight. All subsequent analyses were performed using these normalized weights.

### Data handling and statistical analysis

To account for the multistage sampling structure, a complex survey design object was created using the *svydesign(*) function from the *survey* package. The design specified clustering at the enumeration area level (communities), stratification by sub-district, and applied post-stratification weights derived from the sampling frame. The setting *survey.lonely.psu = “adjust”* was specified to handle strata with only one primary sampling unit (e.g., Torkor). Descriptive analysis was first conducted to estimate weighted distributions and vaccine acceptance proportions across key covariates. For each explanatory variable presented in Table 2, weighted frequencies and proportions were computed using *svytable()* and *prop.table()*, while vaccine acceptance estimates were generated using *svyby()* in conjunction with *svymean()*. A custom function iterated through all candidate variables to produce standardized summaries with 95% confidence intervals, accounting for the survey design.

**Table 2:** Univariate Associations Between Sociodemographic, Environmental, and Psychosocial Characteristics and RTS,S/AS01 Malaria Vaccine Acceptance.

Variables	Weighted n [%]	RTS,S vaccine acceptance [95% CI]	cOR (95% CI)	P-value
Respondents age				
< 20	2577 [43.82]	92.37 [79.52 - 97.42]	1	
20-24	1328 [22.59]	87.57 [72.68 - 94.91]	0.94 [0.41, 2.16]	0.876
25-29	894 [15.19]	83.03 [63.15 - 93.32]	0.55 [0.17, 1.74]	0.288
≥ 40	1082 [18.40]	92.80 [82.36 - 97.27]	0.38 [0.11, 1.26]	0.108
Respondents sex				
Male	1654 [28.12]	90.65 [76.90 - 96.58]	1	
Female	4228 [71.88]	89.67 [77.93 - 95.52]	0.90 [0.36, 2.20]	0.800
Religion				
Christian	5708 [97.05]	89.91 [78.59 - 95.58]	1	
Muslim	174 [2.95]	91.02 [73.74 - 97.34]	1.39 [0.35, 5.55]	0.627
Household size				
One to three	1572 [26.73]	91.58 [81.68 - 96.37]	1	
Four	1287 [21.88]	83.88 [64.30 - 93.76]	0.48 [0.17, 1.38]	0.161
Five	1190 [20.23]	92.11 [79.52 - 97.23]	1.07 [0.44, 2.62]	0.872
Six and above	1833 [31.16]	91.40 [76.78 - 97.16]	0.98 [0.25, 3.84]	0.972
Residential status				
Urban	187 [3.17]	97.59 [69.61 - 99.86]	1	
Rural	5695 [96.83]	89.70 [78.86 - 95.31]	0.21 [0.01, 3.21]	0.249
Mother’s education				
Tertiary	490 [8.32]	89.94 [62.46 - 97.96]	1	
Secondary	3226 [54.84]	92.53 [84.52 - 96.57]	1.39 [0.35, 5.55]	0.627
Basic	1842 [31.31]	84.49 [60.17 - 95.16]	0.61 [0.12, 3.16]	0.535
None	325 [5.53]	95.20 [80.01 - 98.99]	2.22 [0.17, 29.28]	0.524
Father’s education				
Tertiary	725 [12.32]	87.45 [67.07 - 95.97]	1	
Secondary	3474 [59.06]	91.96 [80.59 - 96.93]	1.64 [0.73, 3.71]	0.217
Basic	1420 [24.15]	85.52 [58.66 - 96.09]	0.85 [0.14, 5.16]	0.850
None	263 [4.47]	94.07 [75.97 - 98.76]	2.28 [0.27, 19.43]	0.430
**Mother’s occupation**				
Farming	732 [12.44]	99.36 [94.64 - 99.93]	10.40 [1.00, 107.83]	0.050
Fish mongering	514 [8.73]	94.76 [82.18 - 98.61]	1.16 [0.37, 3.65]	0.780
Hairdressing	175 [2.98]	94.12 [63.58 - 99.32]	1.03 [0.07, 15.11]	0.982
Nursing	182 [3.10]	95.08 [68.15 - 99.82]	1.24 [0.10, 16.03]	0.858
Seamstress	285 [4.85]	98.42 [87.31 - 99.82]	4.02 [0.46, 35.07]	0.190
Teaching	369 [6.27]	85.06 [61.32 - 95.34]	0.37 [0.16, 0.83]	**0.020**
Trading	2677 [45.51]	84.17 [68.04 - 92.99]	0.34 [0.13, 0.92]	**0.035**
Unemployed/Others/Student	949 [16.13]	93.96 [87.61 - 97.28]	1	
Father’s occupation				
Farming	1850 [31.46]	96.53 [88.15 - 99.05]	1.87 [0.68, 5.17]	0.208
Fishing	553 [9.41]	70.22 [18.21 - 96.15]	0.16 [0.01, 2.14]	0.152
Carpentering/Masoning	406 [6.90]	75.61 [34.26 - 94.85]	0.21 [0.03, 1.38]	0.097
Tricycle (Okada) riding/Driving	946 [16.08]	93.55 [85.23 - 97.33]	0.97 [0.35, 2.70]	0.958
Teaching	527 [8.95]	83.59 [60.76 - 94.37]	0.34 [0.10, 1.19]	0.088
Trading	502 [8.53]	90.68 [79.26 - 96.12]	0.65 [0.24, 1.80]	0.387
Others **	1098 [9.09]	93.70 [87.45 - 99.95]	1	
**Marital status**				
Married	2453 [41.70]	83.18 [64.46 - 93.10]	0.27 [0.09, 0.79]	0.020
Single/cohabiting	3429 [55.79]	94.55 [87.87 - 97.65]	1	
Average monthly income				
< 1000.00	1420 [24.13]	96.85 [89.53 - 99.10]	1	
1000 – 1400	405 [6.88]	82.07 [54.76 - 94.54]	0.15 [0.03, 0.81]	**0.029**
1500 – 1900	892 [15.17]	70.62 [41.50 - 89.06]	0.08 [0.02, 0.36]	**0.003**
2000 – 2400	784 [13.33]	85.53 [40.47 - 97.17]	0.19 [0.02, 1.99]	0.155
>= 2500	2381 [28.45]	95.87 [89.96 - 98.74]	0.76 [0.09, 6.45]	0.786
Number of children under 5 years in household				
One	5126 [87.15]	90.70 [79.76 - 96.02]	1	
Two	630 [10.71]	92.23 [69.99 - 98.38]	1.22 [0.24, 6.20]	0.802
Three to four	126 [2.14]	47.63 [7.23 - 91.38]	0.09 [0.01, 0.89]	**0.040**
Age of youngest child				
Four	1049 [17.83]	93.40 [84.67 - 97.31]	1	
Under One	2185 [37.16]	91.06 [79.72 - 96.35]	0.72 [0.27, 1.94]	0.495
One	1527 [25.96]	93.70 [82.94 - 97.85]	1.05 [0.41, 2.70]	0.911
Two	1121 [19.06]	79.43 [61.59 - 90.29]	0.27 [0.11, 0.67]	**0.007**
Sex of youngest child				
Male	2672 [45.43]	86.81 [70.84 - 94.69]	1	
Female	3210 [54.47]	92.56 [83.46 - 96.84]	1.89 [0.72, 4.94]	0.183
Building has standing water around the house				
No	3875 [65.88]	86.74 [72.76 - 94.13]	1	
Yes	2007 [34.12]	96.12 [88.31 - 98.79]	3.79 [1.07, 13.45]	**0.040**
Building has no windows or is covered with cloth				
No	5002 [85.03]	90.43 [79.21 - 95.91]	1	
Yes	880 [14.97]	87.20 [65.66 - 96.02]	0.72 [0.21, 2.44]	0.582
Windows has no mosquito screens				
No	4329 [73.6]	90.00 [77.16 - 95.99]	1	
Yes	1553 [26.40]	89.81 [78.35 - 95.55]	0.98 [0.38, 2.53]	0.964
House surrounded by bushes				
No	2039 [34.66]	79.57 [56.08 - 92.23]	1	
Yes	3843 [65.34]	95.45 [90.56 - 87.87]	5.39 [2.01, 14.48]	**0.002**
No	2281 [38.78]	91.49 [81.22 - 96.39]	1	
Yes	3601 [61.22]	88.97 [74.13 - 95.78]	0.75 [0.26, 2.19]	0.582
Do you have mosquito bed net?				
Yes	5606 [99.31]	92.25 [81.51 - 96.98]	0.06 [0.01, 0.67]	**0.024**
No	276 [4.69]	43.06 [9.03 - 85.20]	1	
Do you have health insurance?				
Yes	5718 [97.20]	90.11 [79.62 - 95.51]	0.58 [0.13, 2.64]	0.466
No	164 [2.80]	84.18 [43.10 - 97.39]	1	
Have you heard of the RTS,S malaria vaccine?				
Yes	2002 [34.04]	96.35 [88.26 - 98.93]	0.25 [0.09, 0.65]	**0.007**
No	3880 [65.96]	86.64 [72.56 - 94.08]	1	
Does the vaccine give full protection against malaria?				
No	4000 [68.01]	86.71 [71.89 - 94.43]	1	
Yes	1882 [31.99]	96.83 [91.42 - 98.87]	4.68 [1.27, 17.33]	**0.023**
Does taking the vaccine means ignoring existing malaria preventive measures?				
No	2842 [48.31]	82.51 [63.93 - 92.62]	1	
Yes	3040 [51.69]	96.90 [93.59 - 98.52]	6.62 [2.44, 17.98]	**0.001**
Do you trust that the malaria vaccine would help prevent malaria?				
No	1656 [28.15]	84.04 [52.73 - 96.13]	1	
Yes	4226 [71.85]	92.26 [83.08 - 96.66]	2.26 [0.41, 12.42]	0.329
Are you comfortable with the routh of administration?				
No	2836 [48.22]	82.83 [64.43 - 92.78]	1	
Yes	3046 [51.78]	96.57 [88.35 - 99.05]	5.83 [1.34, 25.44]	**0.021**
Are you comfortable with the number of doses?				
No	3802 [64.63]	86.38 [71.98 - 93.99]	1	
Yes	2080 [35.37]	96.47 [91.00 - 98.66]	4.31 [1.48, 12.53]	**0.010**
Are you afraid of adverse effects following vaccination?				
No	4313 [36.19]	83.12 [64.46 - 93.03]	1	
Yes	7604 [63.81]	93.82 [86.84 - 97.22]	3.09 [1.79, 5.34]	**0.000**
Can the child’s father’s decision prevent the child from taking the vaccine?				
No	1277 [21.71]	85.59 [58.22 - 96.33]	1	
Yes	4605 [78.29]	91.09 [82.45 - 95.70]	1.69 [0.50, 5.70]	0.379
Overall trust in the vaccine				
No	4165 [70.81]	92.46 [83.68 -96.70]	1	
Yes	1717 [29.19]	83.86 [53.99 - 95.83]	0.42 [0.08, 2.13]	0.281
I will accept the vaccine based on it’s efficacy				
Yes	5154 [87.62]	92.59 [84.70 - 96.58]	0.20 [0.06, 0.62]	**0.008**
No	728 [12.38]	71.24 [39.20 - 90.49]	1	
I will accept the vaccine based on it’s safety				
Yes	4992 [84.88]	94.18 [88.61 - 97.12]	0.12 [0.05, 0.30]	**0.000**
No	890 [15.12]	66.18 [37.99 - 86.21]	1	
I will accept the vaccine by dictates of my religion				
No	5700 [96.90]	89.79 [78.66 - 95.45]	1	
Yes	182 [3.10]	94.71 [79.66 - 98.79]	2.03 [0.43, 9.52]	0.349
I will accept the vaccine based on influence from family or friends				
Yes	4510 [76.67]	93.42 [844.78 - 97.31]	0.26 [0.08, 0.86]	**0.029**
No	1372 [23.33]	78.52 [51.13 - 92.74]	1	
I will accept the vaccine based on healthcare worker providing adequate information				
N	1312 [22.30]	86.92 [59.79 - 96.74]	1	
Yes	4570 [77.70]	90.81 [81.60 - 95.66]	1.49 [0.40, 5.49]	0.532
I will accept the vaccine based on healthcare worker’s attitude towards patients				
No	1488 [25.29]	88.01 [60.04 - 97.39]	1	
Yes	4394 [74.71]	90.60 [81.61 - 95.44]	1.31 [0.32, 5.45]	0.694
Distance to the vaccination centre/point				
Yes	4223 [71.79]	95.26 [89.85 - 97.85]	0.16 [0.06, 0.41]	**0.001**
No	1659 [28.21]	76.42 [53.54 - 90.12]	1	
Availability of adequate information for decision making				
Yes	4448 [75.62]	94.17 [89.19 - 96.94]	0.21 [0.08, 0.55]	**0.003**
No	1434 [24.38]	76.84 [47.94 - 92.28]	1	
Availability of prompts and reminders				
Yes	4052 [68.88]	94.35 [88.51 - 97.30]	0.24 [0.11, 0.53]	**0.001**
No	1830 [31.12]	80.23 [56.37 - 92.73]	1	
If I am vulnerable to the risk of disease				
Yes	4742 [80.61]	93.17 [85.43 - 96.94]	0.24 [0.06, 1.02]	0.053
No	1140 [19.39]	76.55 [41.48 - 93.76]	1	
Have you had negative experience with vaccine in the past?				
No	4271 [72.61]	87.09 [72.77 - 94.45]	1	
Yes	1611 [27.39]	97.52 [92.00 - 99.26]	5.83 [1.47, 23.18]	**0.015**

Weighted percentages, 95% confidence intervals (CI) of acceptance, and crude odds ratios (cOR) from survey-weighted logistic regression models are shown. ** (Student, Electrician, Auto-Mechanic). Bolded results indicate statistical significance at p < 0.05.

Univariate logistic regression models were then fit using *svyglm()* to evaluate crude associations between each predictor and RTS,S/AS01 vaccine acceptance. Variables with p-values below 0.10 were retained for multivariable analysis. Although multicollinearity diagnostics using the variance inflation factor (VIF) did not indicate problematic correlations among predictors, convergence issues arose during Bayesian hierarchical modeling. To address this, four thematic composite variables were constructed by summing conceptually related binary items, thereby reducing model dimensionality while preserving theoretical meaning. Each composite was standardized (mean = 0, SD = 1) for interpretability. The **Risk Concern Score** included: (1) fear of adverse effects following vaccination, (2) the belief that taking the vaccine implies disregarding other malaria preventive strategies, and (3) prior negative vaccine experience. The **Confidence Score** combined (1) belief that the vaccine is effective, and (2) belief that it is safe. The **Awareness Score** reflected (1) whether the respondent had heard of the RTS,S vaccine, and (2) belief that it offers full protection. The **Perceived Convenience Score** captured (1) comfort with the route of administration, and (2) comfort with the number of required doses. These composite scores were used only in the final multivariable mixed-effects models and not in univariate or descriptive analyses.

Multivariable analysis was conducted using Bayesian multilevel logistic regression via the *brms* package, incorporating a random intercept for sub-district to account for clustering. Six models were estimated: Model 0 (intercept-only) and Models 1–4 (thematic models for sociodemographic, household/environmental, psychosocial, and health system access variables), followed by Model 5 (fully adjusted). Weakly informative priors were specified: *normal(0, 1)* for fixed effects and *student_t(3, 0, 1)* for group-level standard deviations. All models were estimated via Hamiltonian Monte Carlo, and *save_pars = save_pars(all = TRUE)* was used to retain posterior samples. Model fit was assessed using leave-one-out cross-validation (LOO-CV) through the *loo* package, with moment matching applied for observations with high Pareto-k values. Model 5 showed the best fit based on the lowest expected log predictive density (LOO-ELPD) and highest Bayesian R² (0.63). Final inferences were drawn from this model. Adjusted odds ratios (aORs) with 95% credible intervals were reported for all predictors, and intraclass correlation coefficients (ICCs) was computed to quantify the proportion of variance due to sub-district-level clustering. All analyses were conducted in R using reproducible workflows and visualization tools from *tidybayes*, *broom.mixed, bayesplot*, and *survey.*

### Spatial analysis

**Fig 1:**
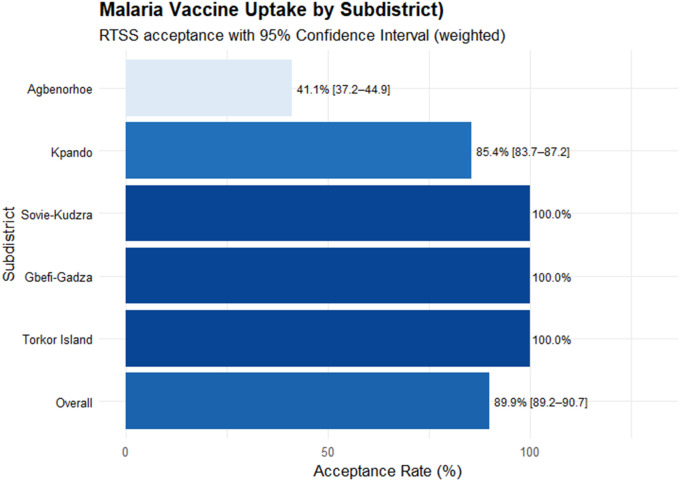
Distribution of survey-weighted RTS,S/AS01 malaria vaccine by sub-districts in the Kpando Municipality. Confidence intervals reflect survey-weighted estimates accounting for clustering and sampling design.

**Fig 2:**
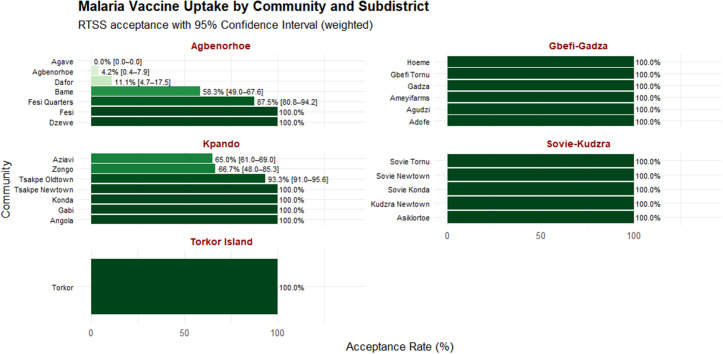
Distribution of survey-weighted RTS,S/AS01 malaria vaccine by communities within sub-districts in the Kpando Municipality. Confidence intervals reflect survey-weighted estimates accounting for clustering and sampling design.

To assess geographic variation in RTS,S/AS01 vaccine acceptance, spatial data were incorporated using shapefiles for the Kpando Municipal Assembly obtained from GADM database (Global Administrative Areas through https://gadm.org/data.html). Administrative boundaries, roads, rivers, and Volta Lake were loaded as vector layers in R using the *sf* and *ggplot2* packages. Spatial objects were transformed to a unified coordinate reference system (UTM Zone 31N), and communities and survey points were geocoded using GPS coordinates. The lake polygon was clipped to municipal boundaries using *st_intersection()*, and all layers were visualized with *geom_sf()* to generate high-resolution maps. These maps facilitated the identification of community-level heterogeneity and spatial clusters of low acceptance. Additionally, weighted bar charts were constructed at both the community and sub-district levels. These plots (Figs 1 and 2) displayed acceptance rates with confidence intervals, stratified by sub-district and annotated to highlight spatial disparities.

### Ethics approval and consent to participate

Ethical clearance for this study was obtained from the Ghana Health Service Ethics Review Committee (GHS-ERC), under protocol number GHS-ERC:035/09/23. The research adhered strictly to the ethical standards set forth in the 2013 revision of the Declaration of Helsinki. Prior to participation, all respondents provided informed consent. For minors aged 15–17 years, both verbal assent and documented guardian consent were obtained in compliance with GHS-ERC guidelines. To ensure comprehension, all study procedures, potential risks, and anticipated benefits were communicated in the participants’ preferred local language (Ewe in most cases and Twi). Written consent was obtained from literate participants, while for those unable to read or write, verbal consent was confirmed in the presence of an impartial witness and recorded with a witness signature, as permitted by the ethics protocol. No identifying personal information, such as names or contact details, was collected, and all data were anonymized to safeguard participant confidentiality. The research team prioritized the principles of respect, autonomy, and non-maleficence throughout the process. No adverse physical or psychological effects were reported during or after data collection.

## Results

### Distribution of weighted sample by sub-district and community

Table 1 presents the distribution of the normalized survey weights and corresponding weighted percentages across sub-districts and communities within the Kpando Municipality. The largest proportion of children were recruited from the Gbefi-Gadza sub-district, accounting for 35.8% (n = 2,106) of the total weighted sample. This was followed by Kpando (26.4%, n = 1,551), Torkor Island (15.5%, n = 912), and Sovie-Kudzra (11.8%, n = 693). Smaller community clusters such as Fesi, Konda, Gabi, and Zongo contributed less than 1% each to the total population estimate.

**Table 1:** Distribution of Survey Weights and Estimated Proportions of Children Under Five Years by Sub-District and Community in the Kpando Municipality, Ghana.

Sub-district/Communities	Pooled weight (N)	Weighted %
Total	5882	100
Agbenorhoe	620	10.54
Agave	123	2.09
Agbenorhoe	108	1.84
Bame	108	1.84
Dafor	93	1.58
Dzewe	90	1.53
Fesi	5	0.09
Fesi Quarters	94	1.60
Gbefi-Gadza	2106	35.81
Agudzi	371	6.31
Adofe	366	6.22
Ameyifarms	349	5.93
Gadza	284	4.83
Gbefi Tornu	360	6.12
Hoeme	377	6.41
Kpando	1551	26.37
Angola	486	8.26
Aziavi	537	9.13
Gabi	24	0.41
Konda	19	0.32
Tsakpe Newtown	14	0.09
Tsakpe Oldtown	446	7.58
Zongo	24	0.41
Sovie-Kudzra	693	11.78
Asiklortoe	89	1.51
Kudzra Newtown	156	2.65
Sovie Konda	132	2.24
Sovie Newtown	136	2.31
Sovie Tornu	179	3.04
Torkor Island	912	15.50
Torkor	912	15.50

**Fig 3:**
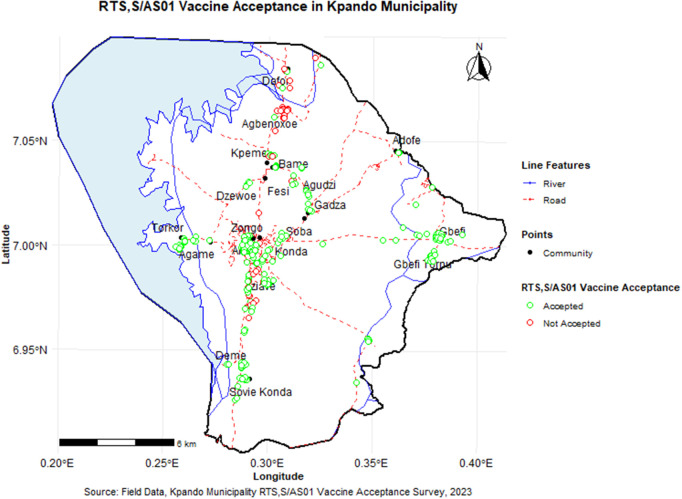
Spatial distribution of RTS,S/AS01 malaria vaccine acceptance across Kpando Municipality. This map visualizes vaccine acceptance rates at the community level. The administrative boundaries used as the base layer were sourced from the Database of Global Administrative Areas (GADM, version 4.1, https://gadm.org/data.html). The terms of use for this data, which permit academic use and publication, are available at https://gadm.org/license.html. Survey data points were georeferenced by GPS coordinates and overlaid on these administrative and environmental layers (roads, rivers, and Volta Lake). Variations in acceptance rates highlight spatial clustering and reveal sub-districts (e.g., Agbenorhoe) with localized low acceptance, guiding targeted intervention planning.

The results indicated an overall RTS,S malaria vaccine acceptance rate of 89.9% (95% CI: 89.2–90.7) across Kpando Municipality. Except for Sovie-Kudzra, Gbefi-Gadza, and Torkor Islands, which recorded universal (100%) acceptance, Kpando town showed an 85.4% acceptance rate, while Agbenorhoe had the lowest at 41.1% (Fig 1). Sub-district analysis revealed that all communities within Sovie-Kudzra, Gbefi-Gadza, and Torkor Islands achieved 100% acceptance. In Kpando town, four out of seven communities reported full acceptance, whereas Tsakpe Oldtown, Zongo, and Aziavi reported acceptance rates of 93.3%, 66.7%, and 65.0%, respectively. Within Agbenorhoe, Fesi and Dzewe communities each recorded 100% acceptance. However, acceptance varied across other communities, ranging from 0.0% in Agave, 4.2% in Agbenorhoe, 11.1% in Dafor, 58.3% in Bame, to 87.5% in Fesi Quarters (Fig 2). The geospacial distribution of the RTS,S/AS01 malaria vaccine acceptance is shown in Fig 3.

### Distribution of RTS,S/AS01 malaria vaccine acceptance by respondents characteristics

Across the entire sample, acceptance of the RTS,S/AS01 malaria vaccine was generally high. Respondents aged <20 and ≥40 reported the highest levels of acceptance at 92.4% and 92.8%, respectively, while those aged 25–29 reported the lowest at 83.0%. Vaccine acceptance was slightly higher among males (90.7%) than females (89.7%) (Table 2). Acceptance rates were also high across religious groups, with Christians (89.9%) and Muslims (91.0%) showing minimal differences. Among household characteristics, those in households with five members (92.1%) had the highest acceptance, while those with four members (83.9%) had the lowest (Table 2).

Interestingly, urban residents reported a notably higher acceptance rate (97.6%) than rural residents (89.7%), despite urban dwellers representing only 3.2% of the weighted sample. Education level showed no consistent pattern; acceptance ranged from 84.5% among mothers with basic education to 95.2% among those with no formal education (Table 2). In terms of occupation, mothers engaged in farming (99.4%), seamstressing (98.4%), and nursing (95.1%) showed the highest acceptance, while those in trading (84.2%) and teaching (85.1%) had comparatively lower rates. For fathers, those engaged in farming (96.5%) and electrician work (95.6%) had higher rates, while those in carpentry/masonry (75.6%) and fishing (70.2%) reported the lowest (Table 2).

Regarding household environment, acceptance was notably higher among those living in homes with standing water nearby (96.1%), surrounded by dense vegetation or overgrown areas (bushes) (95.5%), and those who did not have a mosquito net (43.1%). Awareness and psychosocial variables were strongly linked to acceptance: respondents who had heard of the RTS,S vaccine (96.4%), believed it provides full protection (96.8%), or trusted its safety (94.2%) had much higher rates compared to those who had not (Table 2). Perceptual and attitudinal factors also showed clear differences. Those who were not afraid of adverse effects reported an acceptance rate of 83.1%, while those who were afraid reported 93.8%. Those comfortable with the route of administration (96.6%) or number of doses (96.5%) reported far higher acceptance than those who were not. Similarly, those who perceived availability of information (94.2%), reminders and prompts (94.4%), and influence from family/friends (93.4%) showed significantly greater acceptance than their counterparts (Table 2).

### Univariate regression analysis of factors associated with RTS,S/AS01 malaria vaccine acceptance

Several sociodemographic and environmental variables were significantly associated with RTS,S/AS01 vaccine acceptance. Mothers working in trading (cOR = 0.34, 95% CI: 0.13–0.92, p = 0.035) or teaching (cOR = 0.37, 95% CI: 0.16–0.83, p = 0.020) had significantly lower odds of accepting the vaccine compared to unemployed/others. Lower household income was strongly associated with reduced odds of acceptance; for instance, respondents earning GHC 1500–1900 had 92% lower odds of acceptance (cOR = 0.08, 95% CI: 0.02–0.36, p = 0.003) compared to those earning less than GHC 1000 (Table 2).

From an environmental perspective, homes surrounded by bushes (cOR = 5.39, 95% CI: 2.01–14.48, p = 0.002) and homes with standing water (cOR = 3.79, 95% CI: 1.07–13.45, p = 0.040) were significantly more likely to report vaccine acceptance. Interestingly, ownership of a mosquito bednet was associated with lower odds of acceptance (cOR = 0.06, 95% CI: 0.01–0.67, p = 0.024) (Table 2). Among psychosocial factors, belief in full protection (cOR = 4.68, 95% CI: 1.27–17.33, p = 0.023), confidence in the route of administration (cOR = 5.83, 95% CI: 1.34–25.44, p = 0.021), and confidence in the number of doses (cOR = 4.31, 95% CI: 1.48–12.53, p = 0.010) were significantly associated with higher vaccine acceptance (Table 2). Respondents who believed that taking the vaccine does not mean ignoring other malaria prevention measures were over 6 times more likely to accept the vaccine (cOR = 6.62, 95% CI: 2.44–17.98, p = 0.001). Similarly, prior negative vaccination experience was associated with increased odds of acceptance (cOR = 5.83, 95% CI: 1.47–23.18, p = 0.015) (Table 2).

Table 3 presents the results from six Bayesian multilevel logistic regression models evaluating predictors of RTS,S/AS01 malaria vaccine acceptance. In Model 1, maternal occupation in trading was associated with significantly lower odds of vaccine acceptance (aOR = 0.37, 95% CrI: 0.17–0.84). However, this association attenuated in the full model (Model 5). Environmental factors, particularly the presence of bushes surrounding the household, were strongly associated with acceptance (Model 2: aOR = 4.08, 95% CrI: 2.10–8.35), and remained significant in the full model (aOR = 2.69, 95% CrI: 1.04–6.84). Among psychosocial constructs (Model 3), higher safety perceptions were inversely associated with acceptance (aOR = 0.34, 95% CrI: 0.21–0.54), while higher vaccine acceptance (aOR = 1.62, 95% CrI: 1.16–2.25) and convenience (aOR = 1.62, 95% CrI: 1.03–2.57) were positively associated. These patterns were consistent in the full model, although associations slightly weakened.

**Table 3:** Bayesian Multilevel Logistic Regression Results Predicting RTS,S/AS01 Vaccine Acceptance Across Six Nested Models.

Variables	Model 0 aOR[95%CrI]	Model 1 aOR[95%CrI]	Model 2 aOR[95%CrI]	Model 3 aOR[95%CrI]	Model 4 aOR[95%CrI]	Model 5 aOR[95%CrI]
**Mother’s occupation**						
Farming		2.62 [0.69, 10.92]				2.89 [0.60, 14.01]
Fish mongering		0.63 [0.16, 2.72]				1.31 [0.30, 6.02]
Hairdressing		0.67 [0.15, 3.51]				0.58 [0.11, 2.95]
Nursing		1.69 [0.39, 7.94]				0.87 [0.16, 4.88]
Seamstress		2.27 [0.55, 10.19]				2.47 [0.55, 11.52]
Teaching		0.40 [0.12, 1.38]				0.53 [0.12, 2.12]
Trading		0.37* [0.17, 0.84]				0.43 [0.17, 1.14]
Unemployed/Others		1				1
**Father’s occupation**						
Farming		1.05 [0.39, 2.89]				1.58 [0.49, 5.33]
Fishing		0.27 [0.07, 1.03]				0.45 [0.09, 2.03]
Carpentering/Masoning		0.41 [0.14, 1.21]				0.54 [0.16, 1.82]
Tricycle (Okada) riding/Driving		0.89 [0.33, 2.49]				0.83 [0.27, 2.46]
Teaching		0.66 [0.23, 1.87]				0.59 [0.16, 2.07]
Trading		1.04 [0.36, 2.92]				0.74 [0.21, 2.51]
Others ** (Student, Electrician, Automechanic)		1				
**Marital status**						
Single		1				1
Married		0.78 [0.37, 1.63]				1.88 [0.69, 5.02]
Cohabiting						
**Average monthly income**						
< 1000.00		1				1
1000 - 1400		0.55 [0.18, 1.61]				0.65 [0.18, 2.46]
1500 - 1900		0.91 [0.36, 2.25]				1.12 [0.36, 3.40]
2000 - 2400		1.29 [0.46, 3.62]				1.76 [0.55, 5.71]
≥2500		1.68 [0.65, 4.41]				2.32 [0.80, 6.74]
**Number of children under 5 years in household**						
One		1				1
Two		0.80 [0.29, 2.22]				1.11 [0.32, 3.84]
Three to four		0.85 [0.16, 4.66]				0.69 [0.12, 3.79]
**Age of youngest child**						
Under One		1.29 [0.53, 3.18]				1.19 [0.40, 3.48]
One		1.84 [0.68, 4.99]				1.78 [0.56, 6.01]
Two		1.03 [0.41, 2.63]				1.28 [0.42, 4.14]
Four		1				
**Building has standing water around the house**						
No			1			1
Yes			2.72** [1.22, 6.55]			2.22 [0.78, 6.57]
**House surrounded by bushes**						
No			1			1
Yes			4.08*** [2.10, 8.35]			2.69* [1.04, 6.84]
**Do you have mosquito bednet?**						
Yes			1			1
No			0.44 [0.13, 1.45]			0.69 [0.16, 2.82]
**I will accept the vaccine based on influence from family or friends**						
Yes				2.70** [1.29, 5.65]		1.96 [0.75, 4.90]
No				1		1
**Distance to the vaccination centre/point**						
Yes				0.94 [0.41, 2.09]		0.48 [0.17, 1.36]
No				1		1
**Availability of adequate information for decision making**						
Yes				2.21 [0.91, 5.38]		2.50 [0.93, 6.75]
No				1		1
**Availability of prompts and reminders**						
Yes				1.98 [0.88, 4.42]		1.60 [0.52, 4.84]
No				1		1
Risk concern score					0.34*** [0.21, 0.54]	0.32*** [0.17, 0.58]
Confidence score					1.62* [1.16, 2.25]	1.38 [0.88, 2.15]
Awareness score					1.05 [0.65, 1.69]	1.30 [0.72, 2.37]
Perceived convenience score					1.62* [1.03, 2.57]	1.44 [0.78, 2.68]
**Random Effects**						
Variance	18.632	14.374	13.041	13.103	14.134	18.32
ICC	0.85	0.814	0.799	0.799	0.811	0.848
**Model Fit**						
Log-likelihood	-0.59	-0.08	-0.16	-0.25	-0.40	0.00
LOO-ELPD	-112.22	-107.78	-102.76	-85.51	-100.33	-83.79
**Explained Variance**						
Bayesian R² [95% CrI]	0.37 [0.28 - 0.45]	0.47 [0.40 - 0.53]	0.43 [0.35 - 0.50]	0.56 [0.48 - 0.61]	0.45 [0.37 - 0.51]	0.63 [0.58 - 0.67]

Adjusted odds ratios (aOR) are presented with 95% Bayesian credible intervals (CrI). Asterisks indicate the strength of evidence based on exclusion of the null value (1.0) from the CrI: *** indicates strong evidence (CrI excludes 1.5 or 1/1.5), ** indicates moderate evidence (CrI excludes 1.2 or 1/1.2), and * indicates weak evidence (CrI narrowly excludes 1.0). No asterisk means the CrI includes 1.0, suggesting a non-significant association. Model fit improved with the addition of predictors. The full model (Model 5) demonstrated the best performance with the lowest LOO-ELPD (-83.79) and highest Bayesian R² (0.63, 95% CrI: 0.58–0.67), indicating strong predictive power. Random effect variance and intraclass correlation coefficients (ICC) remained high across models, suggesting substantial between-district variation in vaccine acceptance (Model 5 variance = 18.32, ICC = 0.85).

**Fig 4:**
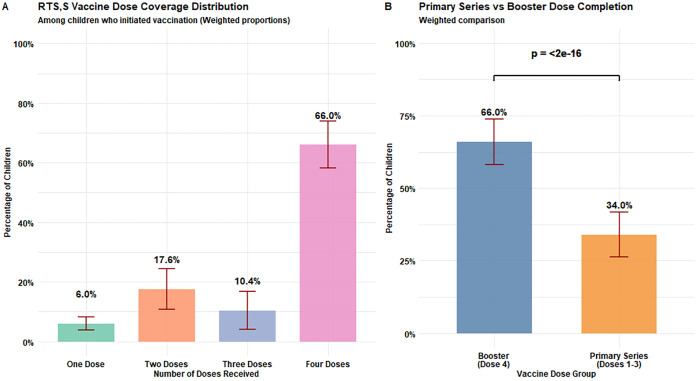
RTS,S Vaccine Dose Coverage Analysis. Panel A: Distribution of RTS,S vaccine dose coverage among children who initiated vaccination, showing weighted proportions for each dose category. Panel B: Comparison of primary series (doses 1-3) completion versus booster (dose 4) completion, with statistical significance indicated. Error bars represent 95% confidence intervals accounting for the complex survey design. All analyses were weighted to reflect the population distribution in Kpando Municipality.

Among children who initiated RTS,S vaccination, dose coverage distribution showed that 6.0% received one dose, 17.6% received two doses, 10.4% received three doses, and 66.0% completed four doses (Fig 4A). In comparing vaccine dose groups, booster dose completion (66.0%) was significantly higher than completion of the primary series (34.0%, p < 2e-16) (Fig 4B).

In the adjusted Bayesian models, household monthly income of 1500–1900 GHC (aOR = 3.19, 95% CrI: 1.34–7.61) and 2000–2400 GHC (aOR = 2.75, 95% CrI: 1.15–6.24) was associated with higher odds of completing the four-dose RTS,S/AS01 schedule compared with households earning <1000 GHC. Caregivers of children aged one year (aOR = 2.27, 95% CrI: 1.13–4.48) and two years (aOR = 2.14, 95% CrI: 1.04–4.40) had higher odds of booster uptake compared with those aged four years. Children from households located near bushes had lower odds of booster uptake (aOR = 0.26, 95% CrI: 0.14–0.49), whereas caregiver reports of family or peer influence (aOR = 2.09, 95% CrI: 1.07–3.97) and perceived convenience of accessing vaccination services (aOR = 1.58, 95% CrI: 1.18–2.12) were associated with higher odds of completing all four doses (Table 4).

**Table 4:** Bayesian Multilevel Logistic Regression Predicting Booster Dose Acceptance Across Six Nested Models.

Variables	Model 0 aOR[95%CrI]	Model 1	Model 2	Model 3	Model 4	Model 5
		aOR[95%CrI]	aOR[95%CrI]	aOR[95%CrI]	aOR[95%CrI]	aOR[95%CrI]
Mother’s occupation						
Farming		0.58 [0.26, 1.32]				0.65 [0.26, 1.55]
Fish mongering		1.87 [0.73, 4.93]				2.48 [0.95, 6.59]
Hairdressing		2.41 [0.75, 8.34]				2.84 [0.83, 10.39]
Nursing		1.36 [0.48, 3.94]				1.55 [0.53, 4.76]
Seamstress		1.67 [0.65, 4.42]				1.68 [0.64, 4.55]
Teaching		1.00 [0.44, 2.44]				0.85 [0.33, 2.23]
Trading		1.21 [0.69, 2.14]				1.19 [0.66, 2.11]
Unemployed/Others		1				1
Average monthly income						
< 1000.00		1				1
1000 - 1400		1.53 [0.67, 3.54]				1.51 [0.61, 3.83]
1500 - 1900		3.40*** [1.55, 7.70]				3.19*** [1.34, 7.61]
2000 - 2400		2.79*** [1.31, 5.90]				2.75*** [1.15, 6.24]
≥2500		1.45 [0.86, 2.48]				1.43 [0.79, 2.59]
Age of youngest child						
Under One		1.24 [0.69, 2.21]				1.58 [0.84, 2.94]
One		1.74 [0.91, 3.35]				2.27*** [1.13, 4.48]
Two		1.98*** [1.01, 3.87]				2.14*** [1.04, 4.40]
Four		1				1
Building has standing water around the house						
No			1			1
Yes			0.67 [0.39, 1.14]			0.65 [0.36, 1.17]
House surrounded by bushes						
No			1			1
Yes			0.27* [0.16, 0.48]			0.26* [0.14, 0.49]
Do you have mosquito bed net?						
Yes			1.31 [0.37, 4.49]			1.48 [0.40, 5.37]
No			1			1
I will accept the vaccine based on influence from family or friends						
Yes					2.32*** [1.27, 4.32]	2.09*** [1.07, 3.97]
No					1	1
Distance to the vaccination centre/point						
Yes					1.58 [0.85, 2.91]	1.28 [0.64, 2.53]
No					1	1
Availability of adequate information for decision making						
Yes					1.06 [0.55, 2.08]	1.14 [0.54, 2.39]
No					1	1
Availability of prompts and reminders						
Yes					0.87 [0.50, 1.49]	0.89 [0.49, 1.60]
No					1	1
Risk concern score				0.96 [0.77, 1.20]		1.04 [0.80, 1.34]
Confidence score				1.19 [0.95, 1.48]		1.11 [0.85, 1.46]
Awareness score				0.81 [0.63, 1.03]		0.96 [0.71, 1.29]
Perceived convenience score				1.19 [0.93, 1.53]		1.58*** [1.18, 2.12]
Random Effects						
Variance	0.944	1.002	0.989	1.12	0.97	1.045
ICC	0.223	0.233	0.231	0.254	0.228	0.241
Model Fit						
Log-likelihood	-0.62	-0.59	-0.59	-0.62	-0.61	-0.56
LOO-ELPD	-241.26	-236.96	-232.02	-242.53	-238.22	-229.22
Explained Variance						
Bayesian R² [95% CrI]	0.073 [0.032 - 0.122]	0.151 [0.101 - 0.201]	0.127 [0.075 - 0.180]	0.109 [0.060 - 0.161]	0.108 [0.061 - 0.159]	0.243 [0.190 - 0.293]

Adjusted odds ratios (aOR) are presented with 95% Bayesian credible intervals (CrI). Asterisks indicate the strength of evidence based on exclusion of the null value (1.0) from the CrI: *** *indicates strong evidence (CrI excludes 1.5 or 1/1.5)*, ** *indicates moderate evidence (CrI excludes 1.2 or 1/1.2)*, and * *indicates weak evidence (CrI narrowly excludes 1.0)*. No asterisk means the CrI includes 1.0, suggesting a non-significant association. Model fit improved with the addition of predictors. The full model (Model 5) demonstrated the best performance with the lowest LOO-ELPD (–229.22) and highest Bayesian R² (0.243, 95% CrI: 0.190–0.293), indicating strong predictive power. Random effect variance and intraclass correlation coefficients (ICC) remained high across models, with Model 5 reporting variance of 1.045 and ICC of 0.241, suggesting substantial between-district variation in vaccine acceptance.

## Discussion

This study represents the first comprehensive assessment of RTS,S/AS01 malaria vaccine acceptance in Kpando Municipality, Ghana, revealing both encouraging trends and significant challenges. The overall acceptance rate of 89.9% demonstrates substantial caregiver confidence in this novel intervention, aligning with rates reported in Ghana’s Upper East Region (94%) [23] and Abuja, Nigeria (98%) [13]. This high baseline acceptance suggests strong community receptivity to malaria prevention innovations in endemic regions. However, the extreme sub-district heterogeneity, ranging from universal acceptance (100%) in Sovie-Kudzra, Gbefi-Gadza, and Torkor Island to critically low acceptance (41.1%) in Agbenorhoe, necessitates urgent investigation into localized barriers. This spatial patterning aligns with findings from Ghana’s Kassena Nankana Municipality, where vaccine coverage varied significantly due to differences in community engagement [23].

Stratification by caregivers’ characteristics revealed unexpected patterns that challenge conventional vaccine delivery frameworks. Contrary to studies emphasizing maternal education [10], acceptance was highest among caregivers without formal education (95.2%) and lowest among those with basic education (84.5%). This paradox may reflect a stronger reliance on community health messaging rather than individual health literacy in decision-making. Similarly, occupational stratification showed that farmers (99.4% acceptance) and seamstresses (98.4%) exhibited higher acceptance than traders (84.2%) and teachers (85.1%), suggesting that vocation-based risk exposure influences prevention behaviors more than socioeconomic status. The inverse relationship between income and acceptance, highest in the lowest income bracket (96.9% for <GHC 1000) and lowest in middle-income groups (70.6% for GHC 1500–1900), warrants exploration of middle-class specific hesitancy drivers, potentially including alternative healthcare access or heightened skepticism about risks.

Environmental determinants proved to be strong predictors of vaccine acceptance. Households surrounded by dense vegetation or overgrown areas (bushes) had significantly higher odds of accepting the vaccine (aOR = 2.69), consistent with malaria transmission patterns in lake-bordering communities where nearby vegetation fosters mosquito breeding [16]. This suggests that caregivers with direct ecological exposure to malaria perceive greater value in vaccination. However, this heightened risk perception coexists with a notable paradox where bed net ownership was associated with markedly lower acceptance (cOR = 0.06). The paradoxical inverse association between bed net ownership and vaccine acceptance may be explained by the distinct distribution channels and perceived roles of these interventions. In Ghana, ITNs are primarily distributed through mass campaigns and antenatal care (ANC) services, decoupling their acquisition from the routine childhood immunization platform where the RTS,S vaccine is delivered. This operational disconnect could foster a perception among caregivers that these tools are alternative, rather than complementary, strategies for malaria prevention. This phenomenon, where the availability of one intervention negatively impacts the uptake of another, has been observed in other health contexts and underscores the challenge of implementing multiple public health measures simultaneously [21]. Our finding highlights a critical need for integrated behavioral messaging and education that explicitly communicates the combined and synergistic benefit of all available malaria prevention tools, including both ITNs and vaccination.

Psychosocial factors, particularly concerns about vaccine safety and information access, provided key insights into their drivers of vaccine acceptance. The strong negative association between vaccine risk concern scores and acceptance (aOR = 0.32) aligns with conventional health behavior models, which suggest that heightened concern about vaccine safety reduces acceptance. Interestingly, despite only 34.0% of caregivers having heard of the RTS,S vaccine, acceptance remained high, pointing to a reliance on implicit trust in the healthcare system rather than informed decision-making. With 78.7% citing health facilities as their main source of information, the influential role of healthcare workers as intermediaries becomes evident. This finding mirrors Ghana’s successful scale-up of IPTp-SP, where clinician advocacy significantly enhanced acceptance [24].

The hierarchical analysis revealed that sub-district random effects accounted for 85% of acceptance variance (ICC = 0.85), dwarfing individual-level predictors. This overwhelming contextual dominance signifies that community-specific factors, historical trust in health systems, local leadership attitudes, or past program implementation quality, determine acceptance more than household characteristics. The Agbenorhoe sub-district exemplifies this, where acceptance ranged from 0% in Agave to 100% in Fesi despite geographic proximity. Such micro-heterogeneity mirrors patterns observed during Ghana’s polio vaccination boycott, where village-specific rumors fueled refusal clusters [12]. This emphasizes the necessity of granular, community-led strategies rather than district-wide approaches.

The lessons learned from the implementation of RTS,S/AS01 extend beyond this specific vaccine. The recent World Health Organization recommendation of the R21/Matrix-M vaccine, with its different efficacy profile and anticipated scale-up, marks a new chapter in malaria prevention [25,26]. However, the success of any malaria vaccine based on findings from this study may, to a very appreciable extent, depend less on modest differences in efficacy but more on the ability to overcome the fundamental barriers identified in this study, vis-à-vis sub-district heterogeneity and caregiver concerns. The strategies proposed, hyperlocal interventions, clear messaging on complementarity, and leveraging trusted health workers, are therefore essential for the equitable and effective introduction of R21 and future malaria vaccines.

Our study revealed a distinctive RTS,S dose distribution pattern characterized by substantial early attrition but strong booster completion (Figure A). While 66.0% of vaccine-initiating children received all four doses, surpassing Ghana’s pilot rollout (50.6%) [27] and Sunyani facility data (about 45%) [28], only 34% completed the primary three-dose series. This inverse attrition pattern, where booster uptake exceeds primary series completion, contrasts with the typical gradual decline observed in routine immunization programs. The finding suggests that children who overcome initial barriers demonstrate strong commitment to completion, yet nearly two-thirds drop out during the primary series. This early attrition aligns with documented challenges in maintaining RTS,S engagement during the second year of life [28,29] and underscores the critical need for targeted interventions during the initial vaccination schedule to improve retention.

Our multivariable analysis identified key determinants of booster-dose acceptance operating across socioeconomic, geographic, and social domains. Economic advantage emerged as a strong predictor: households earning 1500–2400 GHC monthly had 2.7–3.2 times greater odds of full vaccination, consistent with broader evidence that financial barriers substantially increase dropout risk in sub-Saharan Africa [30]. The strong age gradient, with one- and two-year-olds having twice the completion odds of infants, reflects both opportunity accumulation and the concerning pattern of late-dose attrition previously documented in Ghana [29]. Geographic isolation posed a formidable barrier, as residence near bushes (a proxy for rurality) reduced completion odds by approximately 74% (aOR=0.26). Conversely, positive social influences, particularly family/peer encouragement and perceived service convenience, significantly enhanced completion likelihood. These findings collectively emphasize that improving four-dose completion requires addressing both structural constraints (economic and geographic access) while leveraging community-based support systems, suggesting that integrated strategies combining outreach enhancement with social mobilization may be most effective.

This study has several methodological limitations that warrant consideration. First, the cross-sectional design precludes causal inference regarding predictors of vaccine acceptance. Second, the exclusion of 18 hard-to-reach Torkor Island communities, while necessary for feasibility, may limit generalizability to the most remote populations, despite municipal health staff confirming their epidemiological similarity to included clusters. Third, while Bayesian methods robustly handle hierarchical data, the complex model with multiple predictors and random effects requires careful interpretation of credible intervals. Fourth, the composite scores, while addressing multicollinearity, may obscure nuanced relationships between individual psychosocial items and acceptance. Finally, the study did not explore certain potential determinants such as media exposure or detailed household assets, that might provide additional insights into acceptance patterns.

## Conclusion

These findings reveal that Ghana’s malaria vaccine rollout requires a fundamental reorientation from uniform campaigns to precision public health approaches. Our study demonstrates that community-level factors explain 85% of the variance in vaccine acceptance, an overwhelming dominance of contextual over individual determinants. This necessitates three evidence-informed strategies. First, implement hyperlocal interventions targeting specific sub-districts like Agbenorhoe, where community health workers should address location-specific rumors and concerns. Second, leverage the finding that environmental risk exposure (bush-surrounded households) predicts higher acceptance by reframing vaccine messaging to emphasize complementarity with existing tools and targeting areas with lower perceived malaria risk. Third, capitalize on healthcare workers as trusted information conduits through enhanced training on vaccine science and risk communication. The RTS,S/AS01 vaccine represents a transformative opportunity in Ghana’s malaria elimination agenda, but its success hinges on recognizing and addressing the stark sub-district inequities revealed by our Bayesian spatial analysis. By centering community-specific contexts and strengthening frontline health worker capacity, Ghana can transform high aggregate acceptance into equitable, life-saving coverage. These approaches are particularly critical as Ghana prepares for the deployment of additional malaria vaccines, ensuring that implementation strategies are informed by robust local evidence rather than assumed homogeneity.

## Abbreviations

aOR: Adjusted Odds Ratio
cOR: Crude Odds Ratio
CDC: Centers for Disease Control and Prevention
CHPS: Community-based Health Planning and Services
CI: Confidence Interval
CrI: Credible Interval
GHS-ERC: , Ghana Health Service Ethics Review Committee
GSS: Ghana Statistical Service
ICC: Intraclass Correlation Coefficient
ITNs: Insecticide-Treated Nets
LMICs: Low- and Middle-Income Countries
LOO-CV: Leave-One-Out Cross-Validation
LOO-ELPD: Leave-One-Out Expected Log Predictive Density
PPS: Probability Proportion to Size
RTS,S/AS01: Trade name Mosquirix™; a recombinant protein-based malaria vaccine
SD: Standard Deviation
SSA: Sub-Saharan Africa
VIF: Variance Inflation Factor
WHO: World Health Organization.
